# New Helical Binding Domain Mediates a Glycosyltransferase Activity of a Bifunctional Protein*[Fn FN2]

**DOI:** 10.1074/jbc.M116.731695

**Published:** 2016-08-17

**Authors:** Hua Zhang, Meixian Zhou, Tiandi Yang, Stuart M. Haslam, Anne Dell, Hui Wu

**Affiliations:** From the ‡ Departments of Pediatric Dentistry and Microbiology, Schools of Dentistry and Medicine, University of Alabama at Birmingham, Birmingham, Alabama 35294 and; the § Department of Life Sciences, Imperial College London, London, SW7 2AZ, United Kingdom

**Keywords:** adhesin, crystal structure, glycoprotein biosynthesis, glycosylation, glycosyltransferase, Streptococcus

## Abstract

Serine-rich repeat glycoproteins (SRRPs) conserved in streptococci and staphylococci are important for bacterial colonization and pathogenesis. Fap1, a well studied SRRP is a major surface constituent of *Streptococcus parasanguinis* and is required for bacterial adhesion and biofilm formation. Biogenesis of Fap1 is a multistep process that involves both glycosylation and secretion. A series of glycosyltransferases catalyze sequential glycosylation of Fap1. We have identified a unique hybrid protein dGT1 (dual glycosyltransferase 1) that contains two distinct domains. N-terminal DUF1792 is a novel GT-D-type glycosyltransferase, transferring Glc residues to Glc-GlcNAc-modified Fap1. C-terminal dGT1 (CgT) is predicted to possess a typical GT-A-type glycosyltransferase, however, the activity remains unknown. In this study, we determine that CgT is a distinct glycosyltransferase, transferring GlcNAc residues to Glc-Glc-GlcNAc-modified Fap1. A 2.4-Å x-ray crystal structure reveals that CgT has a unique binding domain consisting of three α helices in addition to a typical GT-A-type glycosyltransferase domain. The helical domain is crucial for the oligomerization of CgT. Structural and biochemical studies revealed that the helix domain is required for the protein-protein interaction and crucial for the glycosyltransferase activity of CgT *in vitro* and *in vivo*. As the helix domain presents a novel structural fold, we conclude that CgT represents a new member of GT-A-type glycosyltransferases.

## Introduction

Protein glycosylation is one of the most common post-translational modifications. There are two major types of modifications resulting in *N*-linked and *O-*linked glycosylation, respectively. Both are involved in the regulation of various biological processes (1), and have been implicated in human health and diseases (2).

Serine-rich repeat proteins (SRRPs)^2^ are *O-*linked glycoproteins. They belong to a growing family of bacterial adhesins (3–7). Fimbriae associate protein (Fap1) of *Streptococcus parasanguinis* is the first SRRP identified, and is required for biofilm formation (8–10). Fap1-like proteins are highly conserved in other oral streptococci (11–13) and widespread in pathogenic streptococci and staphylococci (4, 5). For instance, Srr-2 of *Streptococcus agalactiae* is associated with bacterial virulence (5), Hsa of *Streptococcus gordonii* and SraP of *Staphylococcus aureus* contribute to the pathogenesis of infective endocarditis (4, 14). PsrP of *Staphylococcus pneumoniae* mediates frequency of invasive pneumococcal disease (15). Thus Fap1-like SRRPs represent a new family of adhesins that are important in bacterial virulence, but little is known about their biogenesis.

Glycosylation of Fap1 is crucial for bacterial adhesion (16), and biofilm formation (17) and is required for assembly of the fimbriae-like surface structure in *S. parasanguinis* (18). An 11-gene cluster flanking the *fap1* gene locus is essential for Fap1 glycosylation and biogenesis (17). Several glycosyltransferase genes including *gly*, *gtf3*, *dgT1*, and *galT2* are found located upstream of *fap1*, and genes coding for accessory secretion proteins such as SecY2, Gap1, Gap2, Gap3, SecA2, and glycosyltransferase Gtf1, and its chaperone Gtf2 are located downstream of *fap1*. SecY2 and SecA2 encode accessory Sec proteins. SecY2 is essential for the biogenesis of mature Fap1 (19), and SecA2 is important for export of mature Fap1 to the cell wall (20, 21). The glycosylation-associated proteins (Gap1, Gap2, and Gap3) modulate Fap1 secretion and biogenesis (18, 22, 23). A glycosyltransferase enzyme complex, consisting of Gtf1 and its chaperone Gtf2, catalyzes the first step of Fap1 glycosylation, transferring GlcNAc residues to the Fap1 polypeptide (17, 24). Gtf3 mediates the second step by transferring Glc to Gtf1–2-modified Fap1 (25, 26). Our previous studies have determined that N-terminal dGT1 (DUF1792) transfers glucose residues to Gtf1, -2, and -3-modified Fap1 (27, 28). The subsequent step of Fap1 glycosylation is still unknown.

dGT1 of *S. parasanguinis* contains a conserved new glycosyltransferase (DUF1792) at the N terminus and a putative glycosyltransferase at the C terminus (27). In this study, we determined the crystal structure of C-terminal dGT1, and found that the C-terminal dGT1 is a GlcNAc transferase, which possesses a glycosyltransferase activity that is distinct from the N-terminal activity, transferring GlcNAc to the Fap1 substrate modified first by the N-terminal DUF1792 glycosyltransferase. The 2.4-Å x-ray crystal structure reveals a new helix domain that is crucial for both oligomerization and enzymatic activity of CgT. Together, we determine that dGT1 is a bifunctional protein that catalyzes successive steps of glycosylation of Fap1 *in vitro* and *in vivo* in *S. parasanguinis*.

## Results

### 

#### 

##### CgT Plays an Important Role in the Fap1 Glycosylation

**FIGURE 1.**
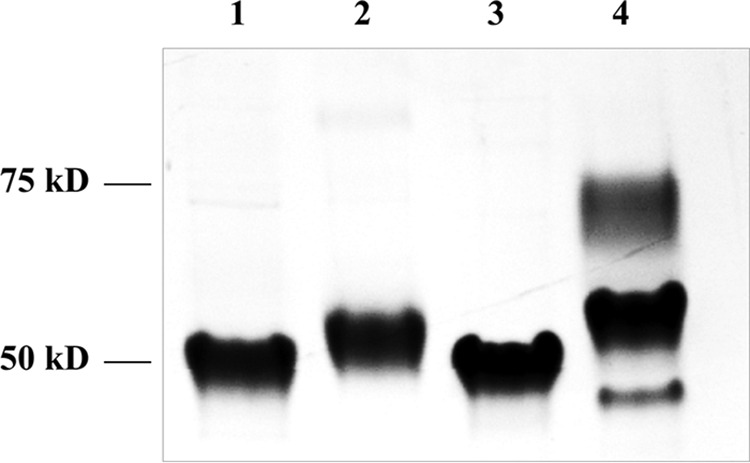
**Glycosylation of rFap1R1 in *E. coli*.** A recombinant small Fap1 fragment (rFap1R1) was modified by Gtf1/2/3 (*lane 1*), Gtf1/2/3 and DUF1792 (*lane 2*), Gtf123 and CgT (*lane 3*), or Gtf1/2/3 and dGT1 (*lane 4*).

dGT1 contains an N-terminal unknown function domain (DUF1792) and C-terminal putative glycosyltransferase domain (CgT). Our studies have shown that DUF1792 is a new type of glycosyltransferases, transferring glucose residues to Glc-GlcNAc-Fap1, and that the C terminus did not have the same activity *in vitro* (27, 28). However, DUF1792 alone failed to complement the dGT1 mutant in *S. parasanguinis* (27), suggesting there is an unknown activity of CgT. To determine the CgT activity, we established an *in vivo* glycosylation system in *Escherichia coli*, using a small recombinant Fap1 (rFap1R1) as a substrate (27). In the system, rFap1R1 was co-expressed with N-terminal DUF1792, C-terminal dGT1 (CgT), or the full-length dGT1 in addition to three glycosyltransferases, Gtf1, Gtf2, and Gtf3. The presence for DUF1792 super-shifted the recombinant Fap1 modified by Gtf123 (Fig. 1, *lane 2 versus 1*). Introduction of the C-terminal domain alone did not modify the recombinant Fap1 glycosylated by Gtf123 (Fig. 1, *lane 3*). When only the full-length dGT1 (Fig. 1, *lane 4*) was used the recombinant Fap1 was further super-shifted to two higher molecular weight positions, indicating that the glycosylation of rFap1RI by DUF1792 is a prerequisite for the subsequent modification by CgT. These data suggest that CgT catalyzes the fourth step of the Fap1 glycosylation.

**FIGURE 2.**
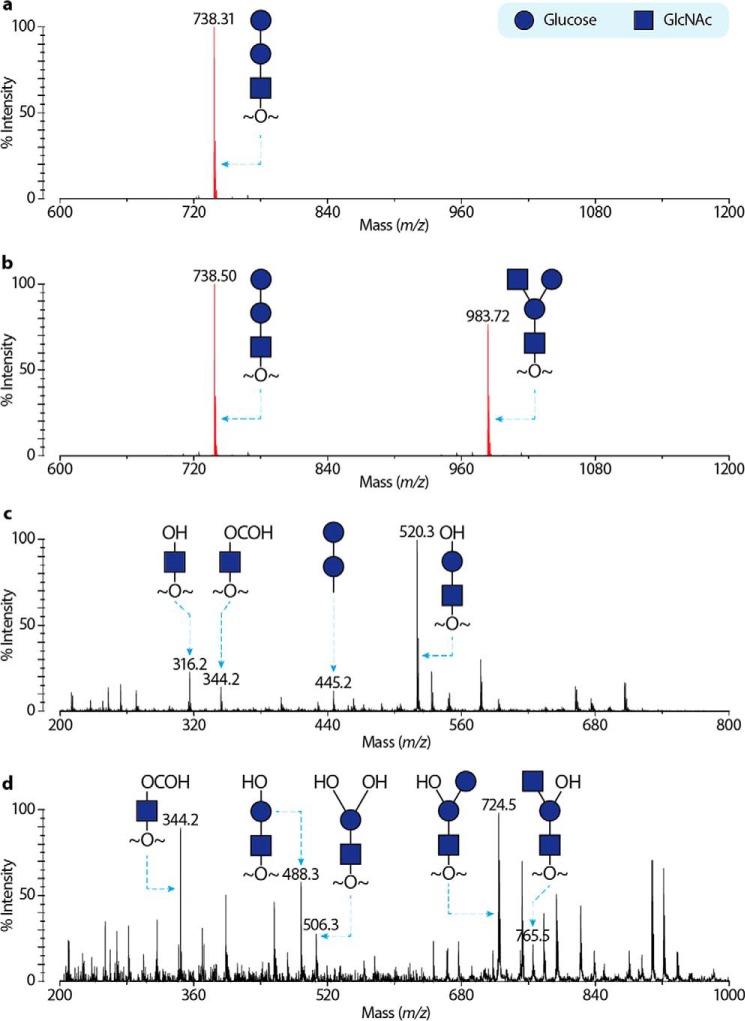
**Mass spectra of DUF1792 and dGT1 modified rFap1.** Glycans from recombinant Fap1 proteins were reductively released and permethylated. Derivatized glycans were purified and analyzed by MS. Representative spectra were shown in *a* (DUF1792 modified rFap1) and *b* (dGT1 modified rFap1). Peaks corresponding to sodiated glycans are colored *red* and annotated with *m*/*z* and glycan structures. *Black* signals are due to under-permethylation (minus 14 in *m*/*z*) or the matrix contaminations. *c,* MS/MS of the MS peak at *m*/*z* 738 from *a*; *d,* MS/MS of the MS peak at *m*/*z* 983 from *b*.

To investigate glycan residue(s) transferred by CgT, we performed MS-based glycomics. The recombinant Fap1R1 proteins glycosylated by DUF1792 and dGT1 were purified and subjected to reductive elimination to release *O-*linked glycans. The isolated glycans were derivatized and subsequently analyzed by mass spectrometry. Matrix-assisted laser desorption/ionization-time of flight (MALDI-TOF) mass fingerprinting (Fig. 2*B*) of the permethylated glycans released from dGT1- and Gtf123-modified rFap1R1, showed a peak corresponding to a tetrasaccharide comprising two HexNAcs and two hexoses. The sequence of the monosaccharides was further characterized by MALDI-TOF/TOF, which generates fragmentation patterns consistent with annotated glycan structures (Fig. 2). Previous and current studies indicate that DUF1792-modified rFap1R1 (Fig. 2*A*) carries a trisaccharide with a sequence of Glc-Glc-GlcNAc and that rFap1R1 can only carry Glc as the hexose and GlcNAc as the HexNAc. Therefore, the presence of an additional HexNAc in the dGT1-modified glycan suggests that the C-terminal dGT1 catalyzes the transfer of GlcNAc to the Gtf123- and DUF1792-modified Fap1, so we hypothesized that CgT has the activity to transfer GlcNAc.

**FIGURE 3.**
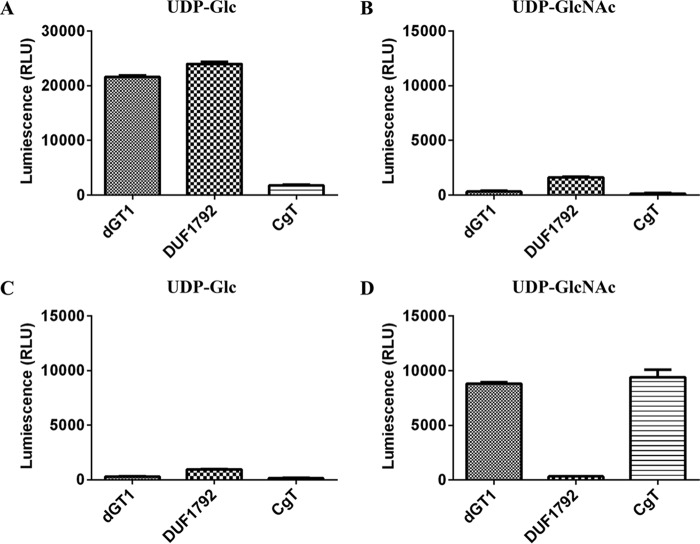
**Glycosyltransferase activities of N terminus (DUF1792), C terminus (CgT), and the full-length dGT1.** *In vitro* glycosyltransferase activity assays were performed using either *Glc-GlcNAc*-Fap1 (*A* and *B*) or *Glc-Glc-GlcNAc*-Fap1 as an acceptor substrate (*C* and *D*). Either UDP-Glc (*A* and *C*) or UDP-GlcNAc (*B* and *D*) was used as a sugar donor in all reactions.

To test this, we performed an *in vitro* glycosyltransferase assay using the UDP-Glo method (Promega) (29). Gtf123-modified Fap1 was used as a substrate, and either UDP-Glc or UDP-GlcNAc was evaluated as a sugar donor. Consistent with our previous data, DUF1792 transfers Glc not GlcNAc residues to Gtf123-modified Fap1 (Fig. 3, *A* and *B*), whereas CgT did not have the activity to transfer either GlcNAc or Glc residues to Gtf123-modified Fap1 (Fig. 3, *A* and *B*). However, when Gtf123- and DUF1792-modified Fap1 was used as a substrate, CgT was able to transfer GlcNAc but not Glc to the substrate (Fig. 3, *C* and *D*). These data are consistent with the findings from the *E. coli* glycosylation system (Fig. 1), demonstrating that CgT is a glycosyltransferase, transferring GlcNAc residues to the Fap1 that is first modified by DUF1792. Taken together, we conclude that CgT mediates the fourth step of Fap1 glycosylation.

##### CT1 Interacts with Itself

**FIGURE 4.**
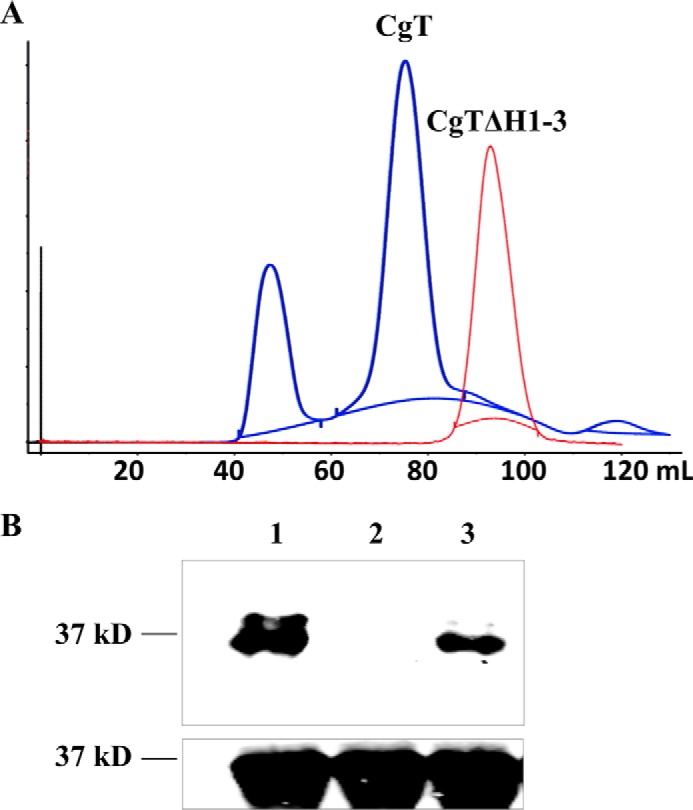
**Gel filtration profile of CgT and CgTΔH1–3 (*A*) and pulldown assay (*B*).** Elution curve of CgT (*blue*) and CgTΔH1–3(*red*) from a GE Hiload 16/60 Superdex G200 column at 4 °C. *B,* GST pull-down of His-tagged CgT. GST fused dGT1 (*lane 1*), DUF1792 (*lane 2*), and CgT (*lane 3*) were used to pull down His-tagged CgT. *Top panel*, His-CgT pulled down by GST fusion proteins, probed by anti-His antibody; *bottom panel*, input control of His-tagged CgT, probed by anti-His antibody.

Gel filtration profile of purified CgT shows two peaks (Fig. 4*A*, *blue line*). The first peak is close to the void volume of this column, SDS-PAGE analysis reveal the presence of an unknown high molecular weight protein (supplemental Fig. 1). The second peak was calculated to accommodate a dimeric CgT, suggesting CgT can bind to itself. An *in vitro* pulldown assay was used to validate this. GST-dGT1 and GST-CgT were able to pull down His-CgT (Fig. 4*B*, *lanes 1* and *3*), whereas GST-DUF1792 failed to pull down His-CgT (Fig. 4*B*, *lane 2*), demonstrating that CgT interacts with itself.

##### Structural Insights into CgT

**FIGURE 5.**
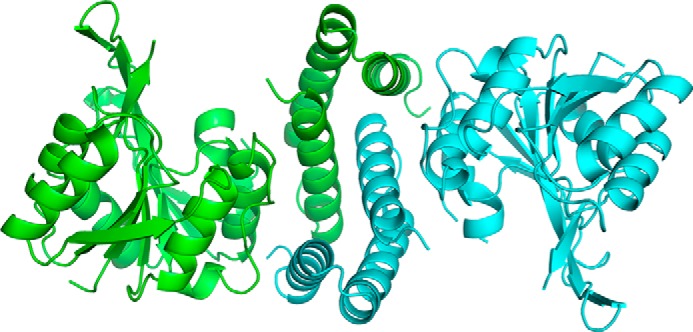
**CgT Structure.** The CgT structure exists in dimer in a space group of R3; each monomer is colored in *green* and *cyan*, respectively. The interface in the CgT structure is the helix domain that faced each other.

To further characterize CgT, we crystallized CgT. Crystals of CgT were obtained at a condition of 0.1 m sodium citrate tribasic dehydrate, pH 5.5, 22% PEG 1000. The x-ray crystal structure of CgT (286–582 amino acids) was determined at 2.4-Å resolution (Table 1). The space group of the CgT structure is R3, shown in dimer in symmetry unit (Fig. 5).

**TABLE 1:** **Data collection and refinement statistics** Statistics for the highest-resolution shell are shown in parentheses.

	CgT
Wavelength (Å)	1.00000
Space group	R3: H
Resolution range (Å)	50.0 − 2.4 (2.5 − 2.4)
Unit cell (Å, °)	*a* = 180.04, *b* = 180.04, *c* = 62.99; α = β = 90.00, γ = 120.00
Total reflections	171513
Unique reflections	29923
Multiplicity	5.70 (5.40)
Completeness (%)	99.86 (98.77)
Mean *I*/σ (*I*)	38.60 (6.20)
Wilson *B*-factor	35.24
CC1/2	(0.84)
*R*_merge_	0.09 (0.46)

**Refinement**	
*R*_work_	0.16 (0.21)
*R*_free_	0.20 (0.25)

**Number of atoms**	4745
Macromolecules	4510
Water	235
Protein residues	552
Root mean square (bonds)	0.009
Root mean square (angles)	1.11
Ramachandran favored (%)	97
Ramachandran outliers (%)	0

**Average *B*-factor**	37.70
Macromolecules	37.40
Solvent	42.70

**PDB code**	5HEC

**FIGURE 6.**
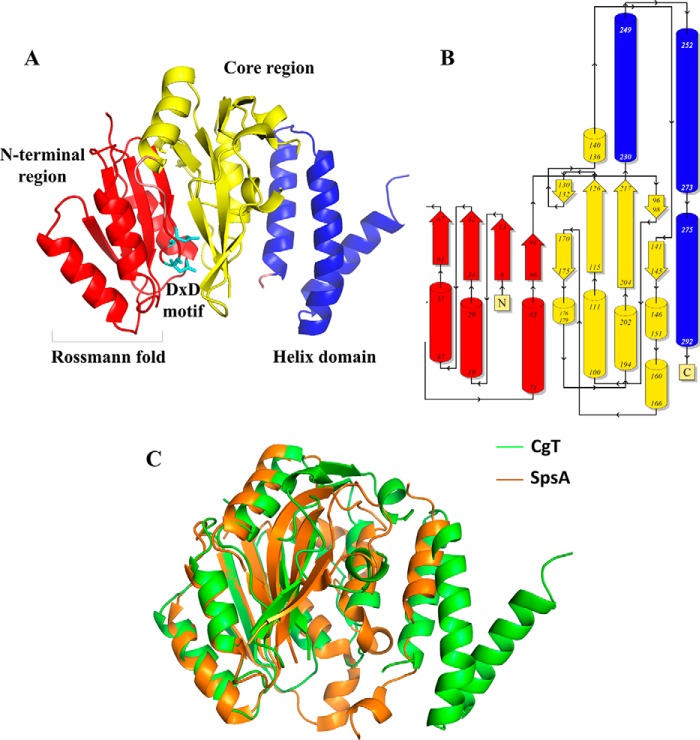
**Structure of the CgT monomer.** The N-terminal region is colored *red*, core region is colored *yellow*, and the helix domain is colored *blue. A,* Rossmann-fold and D*X*D motif are labeled. Topology diagram of the CgT monomer was color coded as the structure. *Arrows* stand for strands, and *columns* represent helices (*B*). Superimposing of CgT with the best matched structure model of SpsA (*C*). CgT is shown in *green*, whereas SpsA is shown in *orange*.

Each CgT monomer has a triangle shape and contains three regions (Fig. 6*A*): an N-terminal domain, a core region, and a helix domain. The N-terminal region consists of β1-β2-β3-β4 and α1-α2-α3. β1, β2, and β3 make a parallel β-sheet surrounded by α1, α2, and α3 to form a Rossmann-fold. Strand β4 connects the N-terminal region with the core of the protein. The core region consists of six strands (β5 to β10) and six helixes (α4 to α9). β7, β8, β9, and β10 lined in the center clamped by α5, α6, and α7 form a sandwich shape (Fig. 6*B*). Two loops (amino acids 154–159 and 219–228) were missing in the core region. At the very C terminus there is a unique helix domain consisting of three helices.

In the CgT structure, two monomers bind tail to tail together through the helix domain (Fig. 5). Because all key binding residues of this CgT dimer were located in the helix domain, we designate it as a helical binding domain.

##### CgT Possesses a Unique Helix Structure

Structural alignment of CgT was performed using the secondary structure matching server from the European Bioinformatics Institute (30). The result shows that only the N-terminal region and part of the core region are matched to SpsA (Protein Data Bank entry 1H7L), the first defined GT-A-type glycosyltransferase (31). SpsA contains a Rossmann-fold and a metal binding site. The match is limited to this region (Fig. 6*C*). We also searched the CgT structure using the Dali server (32). The best match identified is also SpsA. The helix domain does not share any similarity with known glycosyltransferases. The domain was then extracted from the CgT structure, and evaluated using the secondary structure matching server. The best match from the search is TPR2 of FimV (PDB code 4MBQ) from *Pseudomonas aeruginosa* with a low Z score of 3.9. In addition, a Dali search alone revealed it only partially matches bacterial ferritin (FtnA) (PDB code 3R2K) of *P. aeruginosa* with a weak Z score of 5.9. These results suggest the helix domain represents a novel structural fold.

##### The Helical Binding Domain Is Important for the Glycosyltransferase Activity

**FIGURE 7.**
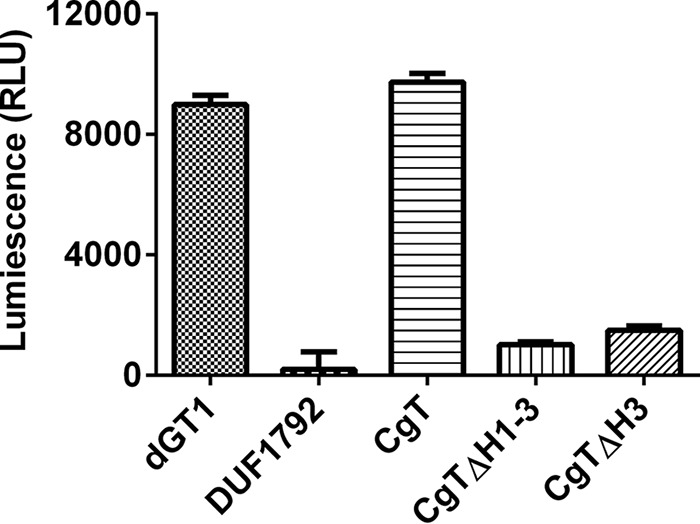
**The helix domain is important for CgT glycosyltransferase activity.** UDP-Glo assay was employed to assess the glycosyltransferase activity of CgT. Gtf123-DUF1792-modified recombinant Fap1 was used as a substrate with UDP-GlcNAc as a sugar donor.

We found all amino acid residues key to the binding distributed along the entire binding interface. Deletion of the entire binding domain from CgT, rendered the CgTΔH1–3 mutant to fail to form a dimer but only exist as a monomer (Fig. 4*A*, *red line*). The CgTΔH1–3 mutant significantly reduced the glycosyltransferase activity *in vitro* (Fig. 7). We also attempted to delete each of three helixes (H1–3) in the binding domain. Interestingly deletion of the first and second helix made CgT unstable and drastically reduced production of CgT (data not shown), which precluded us from studying these deletion mutants. Deletion of the third helix (CgTΔH3) gave rise to a stable protein, the mutant behaved normally but dramatically reduced the glycosyltransferase activity (Fig. 7). These data suggest the helical binding domain is important for the CgT activity.

##### The Helical Binding Domain Does Not Mediate UDP-GlcNAc Binding

**FIGURE 8.**
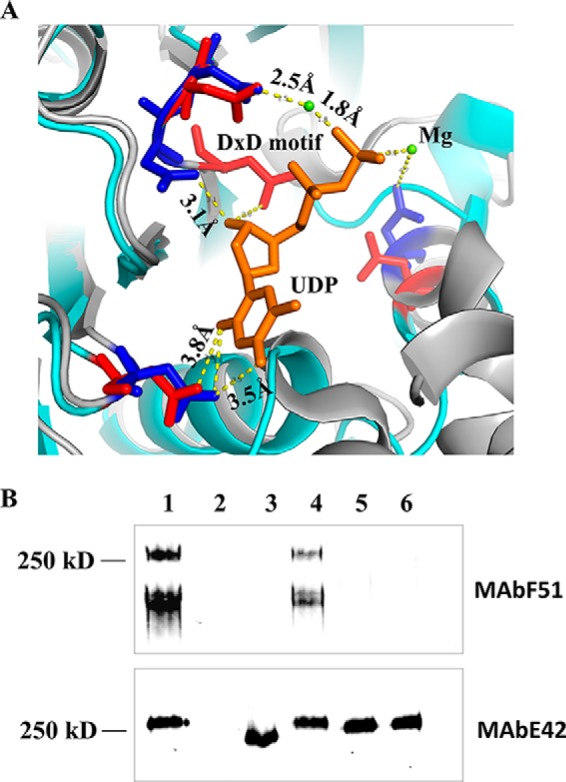
Conserved UDP binding and metal binding sites identified from CgT (*A*). The CgT structure (*green*) superimposed was onto SpsA (*gray*), the key residues in SpsA are colored *blue*, and CgT colored in *red*. UDP, Mg, and the D*X*D motif are labeled. The distance between key residues and UDP is shown as *dotted lines*. The D*X*D motif is important for the CgT function *in vivo* (*B*). Cell lysates of wild type *S. parasanguinis* FW213 (*lane 1*); Fap1 mutant (*lane 2*); dGT1 mutant (*lane 3*); and dGT1 mutant complemented with dGT1 (*lane 4*), dGT1/AXD (*lane 5*), or dGT1/DXA (*lane 6*) were subjected to Western blotting analysis using mature Fap1-specific monoclonal antibody F51 (*top panel*) and Fap1 peptide-specific monoclonal antibody E42 (*bottom panel*) to monitor Fap1 production.

Based on the Dali search, the best matched structure identified is SpsA, a GT-A-type glycosyltransferase. SpsA is a metal-dependent enzyme, containing a D*X*D metal binding motif (33). Structure superimposed between SpsA and CgT revealed the conserved UDP and metal binding residues (Fig. 8*A*). The D*X*D motif is essential for the CgT function, as D*X*D mutants in dGT1 failed to complement dGT1 knock-out *in vivo* (Fig. 8*B*, *lanes 5* and *6*).

**FIGURE 9.**
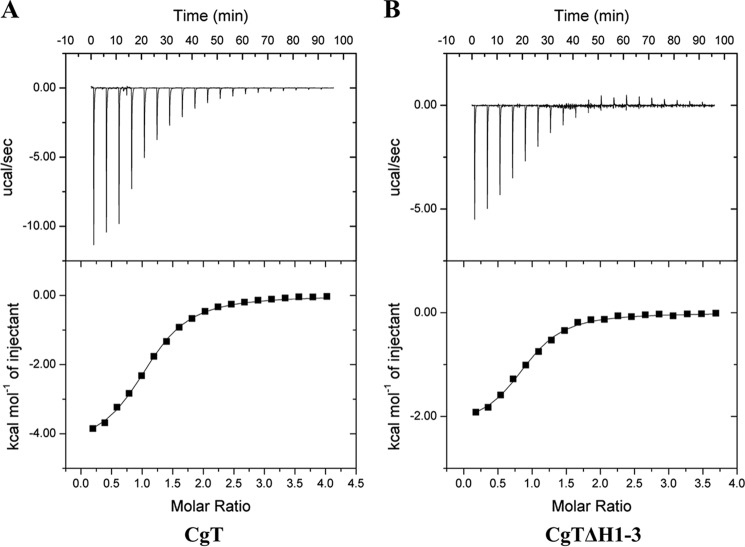
**Isothermal titration calorimetric graphs of CgT and CgTΔH1–3 titrated with UDP-GlcNAc.** Binding of UDP-GlcNAc to CgT or CgTΔH1–3 was assessed as follows. The reaction cells contained either CgT (*A*) or CgTΔH1–3 at 1 mm (*B*) and the syringe was supplied with UDP-GlcNAc at 15 mm. Data obtained from 20 injections of 2-μl aliquots of UDP-GlcNAc at 3-min intervals are shown in the *top graphs*. The *lower plots* show the integrated binding isotherm with the experimental points (■) and best fit.

As the deletion of the helical binding domain reduced the glycosyltransferase activity, and the UDP binding is crucial and close to the helical domain, we also examined whether the helical domain affects the binding of CgT to its substrate UDP-GlcNAc using isothermal titration calorimetry (ITC) (Fig. 9). The *K_d_* value was determined to be 10.67 and 12.15 mm for CgT and CgTΔH1–3 binding, respectively (Table 2), which is much weaker than the reported interactions between glycosyltransferases and their sugar donors. Human α1,4-*N*-acteylhexosaminyltransferase binds to UDP-GlcNAc at *K_d_* of 61 μm (34). Both CgT and CgTΔH1–3 have the same binding ratio to its substrate as being 1:1. The ITC data reveal that both CgT and CgTΔH1–3 bind to UDP-GlcNAc, and the binding affinity is very similar, suggesting the deletion of the helical domain did not affect the binding to UDP-GlcNAc.

**TABLE 2:** **ITC thermodynamic parameters** Δ*H*, Δ*S*, number of binding sites (*n*), and the binding constant (*K_d_*) for donor binding with proteins in solution at 20 °C.

	Cell	Ligand	Number of sites	*K_d_*	Δ*H*	Δ*S*
			*n*	*mm*	*cal/mol*	*cal/mol/deg*
A	CgT	UDP-GlcNAc	1.10 ± 0.02	10.67 ± 1.33	−4358.5 ± 65.5	3.80 ± 0.47
B	CgTΔH1–3	UDP-GlcNAc	0.88 ± 0.01	12.15 ± 1.35	−2148 ± 63	11.45 ± 0.45

##### CgT Is a Distinct Glycosyltranferase and Important for Fap1 Glycosylation in Vivo

**FIGURE 10.**
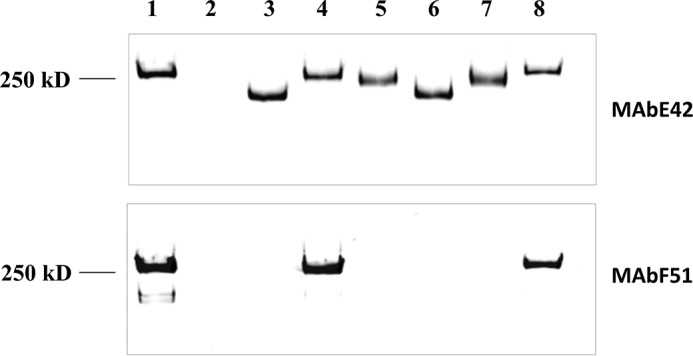
**CgT and the helix domain are required for dGT1 function *in vivo*.** Wild type *S. parasanguinis* FW213 (*lane 1*), Fap1 mutant (*lane 2*), dGT1 mutant (*lane 3*) and its complement variants, dGT1 complemented with the full-length dGT1 (*lane 4*), DUF1792 (*lane 5*), CgT (*lane 6*) with dGT1 without the helix domain (*lane 7*), and with both DUF1792 and CgT (*lane 8*) were examined for the production of Fap1 by Western blotting analysis using Fap1 peptide-specific antibody E42 and mature Fap1-specific antibody F51.

As CgT plays an important role in Fap1 glycosylation *in vitro*, transferring GlcNAc to Glc-Glc-GlcNAc-modified Fap1, we also evaluated its function *in vivo*. N-terminal DUF1792 and C-terminal CgT alone fail to complement the dGT1 mutant in *S. parasanguinis* (Fig. 10, *lanes 5* and *6*), whereas the intact full-length dGT1 complements (Fig. 10, *lane 4*). Intriguingly the split N-terminal DUF1792 and CgT placed on the same plasmid driven by their own promoter was able to complement the dGT1 mutant (Fig. 10, *lane 8*). These data suggest that CgT is an independent glycosyltransferase and important for Fap1 glycosylation. Consistent with the *in vitro* data, the modification of Fap1 by Gtf1, -2, and -3 and DUF1792 is prerequisite for the activity of CgT *in vivo*. DUF1792 partially rescued Fap1 modification, and CgT failed to complement. Only with both DUF1792 and CgT in place was the production of mature Fap1 rescued (Fig. 10, *lane 8*). In addition, dGT1 with deletion of a helical motif also failed to produce mature Fap1 *in vivo* (Fig. 10, *lane 7*), suggesting the importance of the helical domain *in vivo*.

## Discussion

Glycosylation catalyzed by a series of glycosyltransferases is crucial for biogenesis of SRRP adhesins in Gram-positive bacteria (7). dGT1 is a unique bifunctional protein involved in the biosynthesis of an SRRP adhesin Fap1 of *S. parasanguinis*. It is essential for glycosylation of Fap1 by two distinct glycosyltransferase activities derived from the N-terminal and C-terminal regions, respectively. The N terminus of dGT1 was originally annotated as a domain of unknown function (DUF1792) but our recent studies have determined that it represents a new family of glycosyltransferases transferring Glc to Glc-GlcNAc-modified Fap1 (27). In this study we demonstrate that the C terminus of dGT1 (CgT) is an independent glycosyltransferase, transferring GlcNAc to the Glc-Glc-GlcNAc-modified Fap1. The N terminus and C terminus of dGT1 can be separated completely into two enzymes and are still active as long as the split domains are co-expressed. The co-expression fully rescued the dGT1 mutant. On the other hand, the CgT activity depends on the completion of the glycosylation by N-terminal DUF1792. CgT can only glycosylate Fap1 after the Fap1 intermediate is first modified by DUF1792. This claim was substantiated by several pieces of evidence *in vitro* and *in vivo.* 1) The *in vivo E. coli* glycosylation system reconstituted by rFap1 and corresponding glycosyltransferases; 2) mass spectrometric analysis of glycans released from recombinant Fap1 differentially modified by dGT1 or DUF1792; 3) *in vitro* glycosyltransferase reactions using the UDP-GlcNAc sugar donor and the DUF1792-modified Fap1 substrate; and 4) *in vivo* glycosylation of Fap1 in the native host *S. parasanguinis*. Thus we conclude that CgT catalyzes the fourth step of Fap1 following the action of Gtf1/Gtf2, Gtf3, and DUF1792.

The organization of one protein consisting of two glycosyltransferase activities in two separate domains is unique in the SRRP glycosylation pathway. A search of protein databases using dGT1 reveals a number of proteins that share a similar domain organization. This two-domain organization would maximize the enzymatic efficiency because the reaction from one domain should provide the substrate for the subsequent modification by the other domain. Such protein homologs are only found in streptococcal species such as *Streptococcus iniae*, *S. agalactiae*, *Streptococcus suis*, *Streptococcus plurextorum*, *Streptococcus lutetiensis*, *Streptococcus hyovaginalis*, and *Streptococcus gallolyticus* with the exception of *Enterococcus cecorum*. Most of those bacterial genomes contain genes coding for putative SRRP biosynthetic pathways, however, their function and engagement in the biogenesis of SRRPs have not been investigated. Bifunctional glycosyltransferases have been documented in one-domain proteins in eukaryotic cells. *Saccharomyces cerevisiae* β1,4-mannosyl-transferase Alg1 catalyzes the transfer of the first mannose to the glycan during the biosynthesis of asparagine-linked glycoproteins, and the bifunctional mannosyltransferase Alg2, which carries out the dual function of the transfer of two different linkages of mannoses, an α1,3-mannosylation followed by an addition of an α1,6-mannose to complete the first branched pentasaccharide of the glycosylation pathway (35). However, Alg2 only possesses one GT-B-type of glycosyltransferase domain, how the dual activity is executed and regulated is unknown. It is also interesting to note the two-domain dGT1 protein we reported here catalyzes the branching of the glycan sequence (27) as Alg2, how these two domains are coordinated to complete the branched reaction is not clear and awaits further investigation.

A 2.4-Å x-ray crystal structure of CgT revealed another new feature of this glycosyltransferase. CgT contains a unique helix binding domain in addition to possessing a typical GT-A-type glycosyltransferase domain. The helix binding domain is crucial for the oligomerization of CgT and dGT1. The helix binding domain does not appear to mediate the binding of CgT to its sugar donor, UDP-GlcNAc. Furthermore, the deletion of this helical domain significantly reduced glycosyltransferase activity *in vitro* and failed to complement the dGT1 mutant *in vivo*. In addition to the studies of the dual functionality from single domain glycosyltransferases, few distinct two-domain bifunctional glycosyltransferases have been characterized in bacteria (36–40). The hyaluronan synthase of *Pasteurella multocida* (41) and the chondroitin polymerase of *E. coli* K4 (42) are two close examples as they resemble dGT1 and belong to known structural superfamily, GT-A. Capsule polymerases, SiaD_W-135_ and SiaD_Y_, responsible for the biosynthesis of *Neisseria meningitides* capsule polysaccharides, are another unique family of bifunctional glycosyltransferases. The N-terminal GT-B-type glycosyltransferase domain is separated from the C-terminal GT-B-type domain by a structurally unknown center region. They function as galactosyltransferase and sialyltransferase, respectively, however, it is not clear whether the central domain regulates their activities (43). *N*-Acetylglucosamine 1-phosphate uridyltransferase (GlmU) is a cytoplasmic bifunctional enzyme involved in the biosynthesis of the nucleotide-activated UDP-GlcNAc (44). The GlmU structure contains two domains that are linked by a helix arm. The N-terminal domain contains a Rossmann-fold that is involved in the uridyltransferase activity, and C-terminal domain has a left-handed parallel β-helix structure that is responsible for acetyltransferases activity (44). GlmU forms a trimer through the helix arm. Noticeably, this helix arm is not crucial for GlmU activities. This is in sharp contrast to the helix domain of CgT, which is important for enzymatic activity of CgT in addition to its role in the oligomerization of dGT1. Moreover, the helix domain of CgT does not share any sequence and structure homology to any known proteins, and is mostly found in *Streptococcus*, thus representing a unique structure. Because CgT is essential for the Fap1 glycosylation, and the helix domain is a unique binding structure in streptococci, it can be targeted to develop anti-bacteria drugs.

## Experimental Procedures

### 

#### 

##### Strains and Primers

Bacterial strains and plasmids used in this study are listed in Table 3. *E. coli* and *S. parasanguinis* strains were cultured as previously described (45).

**TABLE 3:** **Strains and plasmids used in this study**

Strains or plasmids	Relevant properties	Source
**Strains**		
*E. coli* Top10	Host for propagation of the recombinant plasmids	Invitrogen
*E. coli* BL21-Gold (DE3)	pET system hos strain	Invitrogen
*S. parasanguinis* FW213	Wild type	
*S. parasanguinis* FW213 *Fap1* deletion mutant	Wild type; *Fap1* knockout; *Fap1*::*aphA3*;Kan^r^	8
*S. parasanguinis* FW213 *dGT1* deletion mutant	Wild type; *dGT1* knockout; *dGT1*::*aphA3*;Kan^r^	27

**Strains used to produce recombinant proteins**		
CgT	pET-sumo: CgT transformed into BL21	In this study
His-CgT	pET-28b: CgT transformed into BL21	In this study
CgTΔH1–3	pET-sumo: CgTΔH1–3 transformed into BL21	In this study
CgT-DH1	pET-sumo: CgT-DH1 transformed into BL21	In this study
CgT-DH2	pET-sumo: CgT-DH2 transformed into BL21	In this study
CgT-DH3	pET-sumo: CgT-DH3 transformed into BL21	In this study
His-sFap1-GlcNAc-Glc	pET-28b: sFap1 and pvpt-Gtf123 co-transformed into BL21	27
His-sFap1-GlcNAc-Glc-Glc	pET-28b: sFap1 and pvpt-Gtf123-DUF1792 co-transformed into BL21	In this study
His-sFap1-GlcNAc-Glc(-Glc)-GlcNAc	pET-28b: sFap1 and pvpt-Gtf123-T1 co-transformed into BL21	In this study
GST-DUF1792	pGEx-5x-1: DUF1792 transformed into Top10	27
GST-dGT1	pGEx-5x-1: dGT1 transformed into Top10	27
GST-CgT	pGEx-5x-1: CgT transformed into Top10	In this study

**Plasmids**		
pvpt-hsv-his	*E. coli*-streptococci shuttle vector; Erm^r^	45
pET-sumo	His-SUMO fusion protein expression vector; Kan^r^	25
pET-28b	His fusion protein expression vector; Kan^r^	Amersham
pGEx-6p-1	GST fusion protein expression vector; Amp^r^	Amersham
pET-sumo: CgT	*CgT* cloned in pET-sumo; Kan^r^	In this study
pET-28b: CgT	*CgT* cloned in pET-28b; Kan^r^	In this study
pET-sumo: CgTΔH1–3	Deletion of whole helix domain from pET-sumo: CgT; Kan^r^	In this study
pET-sumo: CgT-DH1	Deletion of first helix domain from pET-sumo: CgT; Kan^r^	In this study
pET-sumo: CgT-DH2	Deletion of second helix domain from pET-sumo: CgT; Kan^r^	In this study
pET-sumo: CgT-DH3	Deletion of third helix domain from pET-sumo: CgT; Kan^r^	In this study
pvpt-dGT1	*dGT1* cloned into pvpt-hsv-his; Erm^r^	In this study
pvpt-DUF1792	*DUF1792* cloned into pvpt-hsv-his; Erm^r^	In this study
pvpt-CgT	*CgT* cloned into pvpt-hsv-his; Erm^r^	In this study
pvpt-DUF1792-pmal-CgT	*CgT* with promoter cloned into pvpt-DUF1792; Erm^r^	In this study
pvpt-Gtf123	*Gtf12* and *Gtf3* cloned into pvpt-hsv-his; Erm^r^	27
pvpt-Gtf123-DUF1792	*Gtf12* and *Gtf3* and *DUF1792* cloned into pvpt-hsv-his; Erm^r^	In this study
pvpt-Gtf123-T1	*Gtf12* and *Gtf3* and *dGT1* cloned into pvpt-hsv-his; Erm^r^	In this study
pvpt-dGT-AxD	Site-direct mutant Asp378 to Ala from pvpt-dGT1; Erm^r^	In this study
pvpt-dGT-DxA	Site-direct mutant Asp380 to Ala from pvpt-dGT1; Erm^r^	In this study
pGEx-6p-1: DUF1972	*DUF1972* cloned into pEGx-6p-1; Amp^r^	27
pGEx-6p-1: dGT1	*dGT1* cloned into pEGx-6p-1; Amp^r^	27
pGEx-6p-1: CgT	*CgT* cloned into pEGx-6p-1; Amp^r^	In this study

Primers used in this study are listed in Table 4. Plasmid isolation, genomic DNA purification, PCR amplification, and purification of PCR products were carried out as described (45). DNA digestion, ligation, and transformation were performed using standard methods.

**TABLE 4:** **Primers used in this study**

Primers	Sequence
CgT-sumo-BamHI-F	5′-GATCAGGATCCATGGATAATGGTGAATTGATT-3′
CgT-sumo-Xho1-R	5′-GATCACTCGAGTTATTTCTCCTTCGGATAATT-3′
CgT-28b-BamHI-F	5′-GATCAGGATCCGATGGATAATGGTGAATTGATT-3′
CgT-28b-XhoI-R	5′-GATCACTCGAGTTATTTCTCCTTCGGATAATT-3′
CgT-pvpt-SalI-F	5′-GATCAGTCGACATGGATAATGGTGAATTGATT-3′
CgT-pvpt-KpnI-R	5′-GATCAGGTACCTTATTTCTCCTTCGGATAATT-3′
CgTΔH1–3-Kpn1-R	5′-GATCAGGTACCATCTAACATTCGAGTTCTAGAAAGT-3′
Deletion-helix1-F	5′-GATCAGTCGACTATCATACTGGATATATCATCTAA-3′
Deletion-helix1-R	5′-GATCAGTCGACCTTCTTGCATCGATGGGCTATGAT-3′
Deletion-helix2-F	5′-GATCAGTCGACAATTTGTTCAGTCAAATCATAGCC-3′
Deletion-helix2-R	5′-GATCAGTCGACGCTTTACGAAATGGTCAAATTGAA-3′
Deletion-helix3-F	5′-GATCAGTCGACAATTTGACCATTTCGTAAAGCGTC-3′
Deletion-helix3-R	5′-GATCAGTCGACTTGATTGAAAATTATCCGAAGGAGAAA-3′
CgTΔH1–3-F	5′-GATCAGTCGACATGATTAGGTTGTTTGAATGGCT-3′
CgTΔH1–3-R	5′-GATCAGTCGACATCTAACATTCGAGTTCTAGAAAGT-3′
Pvpt-promoterKpnI-F	5′-GATCAGGTACCATGAATGCTCATCCGGAATTC-3′
dGT-D378A-F	5′-TAAATATATTACATTTGTGGCTTCAGACGATTTTGTAGAG-3′
dGT-D378A-R	5′-CTCTACAAAATCGTCTGAAGCCACAAATGTAATATATTTA-3′
dGT-D380A-F	5′-TATTACATTTGTGGATTCAGCCGATTTTGTAGAGGAATTCTA-3′
dGT-D380A-R	5′-TAGAATTCCTCTACAAAATCGGCTGAATCCACAAATGTAAT-3′

##### Reductive Elimination and Permethylation

Glycans were reductively eliminated from rFap1R1 variants and purified in a Dowex® column as previously described (46), and the purified glycans were permethylated and further purified according to published methods (46). Briefly, freeze-dried rFap1 samples and 22 mg/ml of potassium borohydride were dissolved in 400 μl of 0.1 m potassium hydroxide solution. The mixture was incubated at 45 °C for 18 h and 5–6 drops of acetic acid was added for quenching the reaction. The solution was loaded onto the 50E-8C Dowex® column and eluted with 5% acetic acid. The collected solution was concentrated and lyophilized. Excessive borates were co-evaporated with 10% methanolic acetic acid.

For the permethylation, sodium hydroxide (3–5 pellets per sample) was crushed in 3 ml of dry dimethyl sulfoxide solvent. The resulting slurry (0.75 ml) and methyl iodine (750 μl) were added to the prepared sample. The mixture was agitated for 30 min and 2 ml of ultrapure water was added with shaking to quench the reaction. The permethylated glycans were extracted with chloroform (2 ml) and washed with ultrapure water twice. Chloroform was removed under a stream of nitrogen. The permethylated glycans were loaded onto a conditioned C18 Sep-Pak® column, washed with 5 ml of ultrapure water, and successively eluted with 3 ml each of 15, 35, 50, and 75% aqueous acetonitrile. Each fraction was collected and lyophilized, and then used for mass spectrometry analysis.

##### Mass Spectrometry

MS data were acquired by using either a Voyager DE-STRTM MALDI-TOF or a 4800 MALDI-TOF/TOF mass spectrometer (Applied Biosystems, Darmstadt, Germany). The latter instrument was also used for acquiring MS/MS data. The MS mode was calibrated with 4700 Calibration standard kit (Applied Biosystems), and the MS/MS mode was calibrated with human fibrinopeptide B (Sigma). The collision energy for CID MS/MS was set to 1 kV, and the collision gas was argon. 2,5-Dihydroxybenzoic acid was used as matrix. Permethylated glycans were dissolved in 10 μl of methanol, and 1 μl of this solution was premixed with 1 μl of matrix and spotted onto the plate for MS analysis.

##### E. coli Glycosylation System

A small fragment of Fap1 (rFap1R1) that contains the first repeat region (100 to 200 amino acid residues of Fap1) was used as a model to reconstitute Fap1 glycosylation in *E. coli* (27). Recombinant BL21 strains were constructed by co-expression of pET28a-rFap1RI (27) with pVPT-Gtf123 (27), pVPT-Gtf123-DUF1792 (27), pVPT-Gtf123-CgT, or pVPT-Gtf123-dGT1 individually, in which rFap1R1 was differentially modified by different combinations of glycosyltransferases. All plasmids were confirmed by PCR and DNA sequencing. Glycosylated rFap1R1 proteins from different recombinant strains were induced, and purified using the same method as described for the recombinant CgT, and were then examined by SDS-PAGE analysis.

##### Protein Expression, Purification, and Crystallization

The DNA fragment coding for the CgT protein (residue 287–582) was cloned into pET-sumo vector with a primer set of CgT-sumo-BamH1-F and CgT-sumo-Xho1-R (Table 2), and expressed in *E. coli* BL21(DE3), purified as previously described (25, 47), and stored at 4 °C in a buffer that contained 20 mm Tris-HCl, pH 8, 100 mm NaCl, and 0.2 mm Tris(2-carboxyethyl)phosphine. Crystallization screens were conducted using the sitting drop vapor diffusion method with a Phoenix crystallization robot on a 96-well Intelli-plate (Art Robbins Instrument). Designed crystallizations were set up manually using the vapor diffusion hanging drop method with 24-well plates. The drops were set up at a 1:1 ratio of protein to mother liquor and incubated at 20 °C. Crystals grown in a well with 0.1 m sodium citrate tribasic dehydrate, pH 5.5, and 22% PEG1000 of IndexHT (Hampton Research) were used to determine the CgT structure.

##### X-ray Data Collection and Processing

Diffraction datasets for CgT (2.4 Å) were collected at the Argonne National Laboratory on a beam station ID22 at 100 K. Paratone-N was used as a cryoprotectant. The datasets were processed by HKL2000 (48).

##### Molecular Replacement and Refinement

Phases for CgT were calculated using the structure of a glycosyltransferase from *Bacteroides fragilis* (PDB code 3BCV) as a starting model by the molecular replacement method using PHENIX software suite (49). Further refinement and model building were performed using the PHENIX software suite and COOT (49, 50). The final CgT structure was obtained through several refinement cycles, and TLS was used for the final refinement.

##### UDP-Glo Assay

UDP-Glo^TM^ assay (Promega) was used to determine *in vitro* glycosyltransferase activity of CgT. His-tagged recombinant Fap1 (amino acid, 100–200) proteins differentially modified by different glycosyltransferases were purified and used as acceptors. Purified dGT1, DUF1792, and CgT were used as enzymes, and either UDP-Glc or UDP-GlcNAc as a sugar donor. A corresponding acceptor, an enzyme, and a sugar donor were reconstituted in the same reaction buffer (20 mm Tris, pH 8.0, 100 mm NaCl, 0.5 mm MnCl), in a total volume of 5 μl and incubated for 2 h. 5 μl of UDP-Glo reagent were added into each mixture to react for 1 h. Luminescence was determined to monitor glycosyltransferase activity.

##### Isothermal Titration Calorimetry

Thermodynamics of binding of enzyme (either CgT or CgTΔH1–3) and ligand UDP-GlcNAc were characterized using MicroCal Auto-iTC200 at 25 °C. Enzymes were dialyzed extensively against 20 mm Tris-HCl buffer, pH 8.0, containing 100 mm NaCl and 20 mm MgCl. Ligand UDP-GlcNAc was dissolved in the same buffer. 400 μl of enzyme solutions (CgT or CgTΔH1–3 at 1 mm) were titrated with 20 injections of the ligand (2 μl of UDP-GlcNAc at 15 mm). Each injection of the ligand lasted 4 s with 300-s intervals between successive injections. Binding isotherms were generated by plotting the corrected heats of binding against the ratio of the ligand to enzymes. Software supplied by the manufacturer (Origin version 7.0 from Microcal Inc.) was used to calculate dissociation constants (*K_d_*), enthalpies of binding (Δ*H*), stoichiometry (*n*), and entropy of binding (Δ*S*).

##### GST Pulldown Assays

To examine *in vitro* self-interaction of dGT1, relevant GST and His-tagged fusion proteins were prepared. Strains carrying plasmids pGEX-6P-1 and pGEX-dGT1/DUF1792/CgT were used for the preparation of GST, GST-dGT1, GST-DUF1792, and GST-CgT fusion proteins, and the strain carrying plasmid pET-28b-CgT was used for the preparation of the His-tagged CgT protein. A primer set of CgT-28b-BamHI-F and CgT-28b-XhoI-R was used to amplify the CgT fragment to construct pET-28b-CgT. A primer set of CgT-sumo-BamHI-F and CgT-sumo-XhoI-R was used to amplify the CgT gene and construct pGEX-6p-1-CgT. Confirmed constructs were used to transform into appropriate *E. coli* strains to produce recombinant proteins.

GST pulldown assay was conducted as described (23, 51). In brief, GST fusion proteins were purified and bound to glutathione-Sepharose 4B beads (Amersham Biosciences) according to the manufacturer's instructions. His-CgT was purified by a His trap column (GE) according to the manufacturer's instructions. 50 μg of glutathione-Sepharose-bound GST fusion proteins were mixed with 40 μg of His-tagged CgT in the NETN buffer (20 mm Tris-HCl, pH 7.2, 100 mm NaCl, 1 mm EDTA, 0.2% Nonidet P-40) and incubated overnight on a rotary shaker at 4 °C. The beads were washed 3 times with 600 μl of NETN buffer and the proteins were eluted with SDS-PAGE sample buffer, boiled for 10 min, and subjected to Western blotting analysis with anti-His antibody at 1:2000 dilution.

##### Complementation of dGT1 Mutant with dGT1 Variants

Shuttle plasmid pVPT-HSV-His was used as a vector to complement the *dGT1* mutant. The DNA fragment corresponding to *CgT* was PCR amplified using a primer pair of CgT-pvpt-sal1-F and CgT-pvpt-Kpn1-R from the genomic DNA of *S. parasanguinis* FW213. The amplified fragment was purified and then cloned into the pVPT-Hsv-His vector to obtain pVPT-CgT. The CgT gene fragment with a functional promoter from pVPT-CgT was amplified using a primer set of pvpt-promoter-kpn1-F and CgT-pvpt-KpnI-R and then cloned into pVPT-DUF1792 to yield pVPT-CgT/DUF172. Plasmid constructs were confirmed by DNA sequencing, and then transformed into the *dGT1* mutant by electroporation as described (27). Transformants were selected on TH agar containing 125 μg/ml of kanamycin and 10 μg/ml of erythromycin, and further verified by PCR analysis. The confirmed strains were used in this study.

##### Site-directed Mutagenesis of the DXD Motif and Truncation of the Helical Binding Domain

Site-directed mutagenesis was carried out using QuikChange mutagenesis kit as described (Stratagene) (25). The plasmid pVPT-dGT1 was used as a template, and two primer sets of dGT-D378A-F and dGT-D378A-R, and dGT-D380A-F and dGT-D380A-R (Table 2) were used to construct the designed mutant alleles. Mutant constructs were identified and confirmed by DNA sequencing, and transformed to the dGT1 mutant to generate dGT1 mutant variants in *S. parasanguinis.*

To construct helical binding domain mutants, plasmid pET-sumo-CgT was used as a template, and the following primer sets, Deletion-helix1-F and Deletion-helix1-R, Deletion-helix2-F and Deletion-helix2-R, Deletion-helix3-F and Deletion-helix3-R, and CgTΔH1–3 and CgT-ΔH1–3-R (Table 2) were used to delete helix1, helix2, helix3, or the entire helix domain, respectively. Mutant alleles were identified and confirmed by PCR and DNA sequencing, and transformed into *E. coli* to induce and express recombinant CgT variants.

## Author Contributions

H. Z., M. Z., S. M. H., A. D., and H. W. designed the study. H. Z. and H. W. drafted the paper. H. Z. and T. Y. performed the experiments. H. Z., T. Y., S. M. H., A. D., and H. W. analyzed the results and wrote the final version of the manuscript.

## Supplementary Material

Supplemental Data

## Footnotes

2 The abbreviations used are:

SRRP: serine-rich repeat protein
ITC: isothermal titration calorimetry
PDB: Protein Data Bank.
