# Supplementary material for: New Helical Binding Domain Mediates a Glycosyltransferase Activity of a Bifunctional Protein
Source: J Biol Chem. 2016 Aug 17;291(42):22106–17. doi: 10.1074/jbc.M116.731695 (PMC5063993; doi:10.1074/jbc.M116.731695)
Supplement: Supplemental Data [file supp_291_42_22106__index.html]

A New Helical Binding Domain Mediates a Unique Glycosyltransferase Activity of a Bifunctional Protein — New Helical Binding Domain Mediates a Glycosyltransferase Activity of a Bifunctional Protein — A New α-Helix Motif Required for Protein Glycosylation — Supplemental Data 

# New Helical Binding Domain Mediates a Glycosyltransferase Activity of a Bifunctional Protein

## Supplemental Data

- supplemental figure 1 (.docx, 257 KB) - Gel filtration profile for CgT.
