# Supplementary material for: New Helical Binding Domain Mediates a Glycosyltransferase Activity of a Bifunctional Protein
Source: J Biol Chem. 2016 Aug 17;291(42):22106–17. doi: 10.1074/jbc.M116.731695 (PMC5063993; doi:10.1074/jbc.M116.731695)
Supplement: Supplemental Data [file 10.1074_M116.731695_jbc.M116.731695-1.docx]

Supplementary information

A new helical binding domain mediates a glycosyltransferase activity of a bifunctional protein

**Hua Zhang^a^, Mei-xian Zhou^a^, Tian-di Yang^b^, Stuart M. Haslam^b^, Anne Dell^b^ and Hui Wu^a^**

^a^Departments of Pediatric Dentistry and Microbiology, Schools of Dentistry and Medicine, University of Alabama at Birmingham, Birmingham, Alabama 35294, USA

^b^Department of Life Sciences, Imperial College London, London, SW7, 2AZ, UK

**Running title**: *A new alpha helix motif required for protein glycosylation*

*Address for correspondence: Prof. Hui Wu, Department of Pediatric Dentistry, University of Alabama at Birmingham, School of Dentistry, 1919 7^th^ Avenue South, Birmingham, AL 35294; E-mail: hwu@uab.edu

**Key words**: Streptococcus, glycoproteins, alpha helix domain, glycosyltransferase, protein-protein interactions, crystal structure

1 2 3


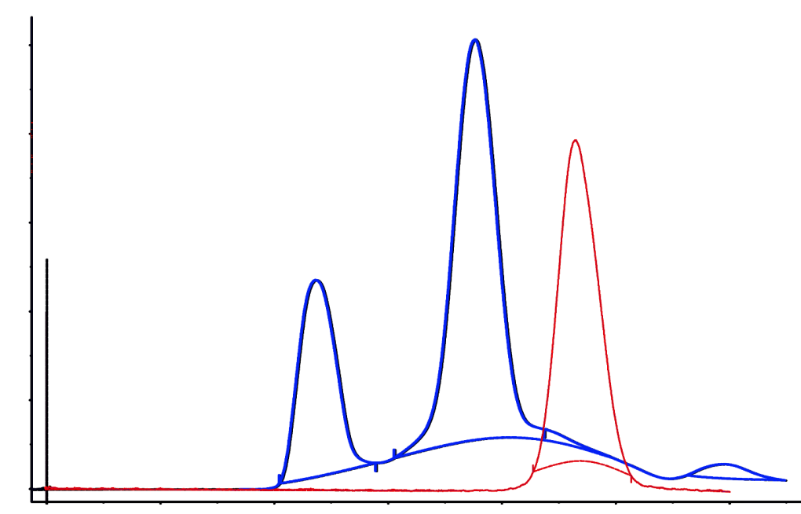


40

60

80

100

120 mL

20

**CgT,**

**Second peak**

**CgTΔH1-3**

**CgT,**

**First peak**





250 kD

37 kD

25 kD

Supplemental figure 1

**Gel filtration profile of CgT and CgTΔH1-3.** Elution curve of CgT and CgTΔH1-3 from GE Hiload 16/60 Superdex G200 column at 4 °C. The blue curve is for CgT, and the red curve stands for CgTΔH1-3 (A). The SDS-PAGE analysis reveals the protein profile for the first peak of CgT (lane 1), the second peak of CgT (lane 2) and CgTΔH1-3 (lane 3) (B). The major bands in the first peak of CgT are protein CgT and a high molecular weight unknown protein (see the black arrow in B); the major protein in the second peak of CgT is CgT. The major band in the peak of CgTΔH1-3 is CgTΔH1-3.
